# Adulterated and Unreliable Drugs

**Published:** 1883-05

**Authors:** 


### ADULTERATED AND UNRELIABLE DRUGS.

Our exchanges point to an enormous evil in the wholesale adulteration of staple drugs both at home and abroad. Quinine sulphate is imported into the Parisian hospitals, in boxes packed at bottom and top with the pure sulphate, while the intermediate space is fraudulently stuffed with a cheaper and inferior salt.

This importation comes from a consolidated Italian and German manufactory at Milan.

A similar complaint is made of the essential oils; very few of these being now found pure and unadulterated. The cheaper oil of turpentine, is most frequently used in this honorable money making process. For the oil of winter-green, the oil of sassafras is the adulterant.

But these examples are only a drop in this sea of manufacturing and commercial fraud. Entire pages could be devoted to its illustration.

We remember a time when the inspection laws of the United States being rigidly enforced at ports of entry against the introduction of the bogus products of these foreign drug manufactories; but not to be outwitted, like the doctors of Alabama, when too hard pressed by the recently formed examining medical boards, suddenly transfer their learning and skill to other and more congenial States. They crossed the Atlantic and established imposing branch manufactories in several United States sea ports. In this way they hoped to escape the penalties that menaced them at home, by getting inside the lines, and behind the batteries which were shelling them from their most lucrative fields of distribution in America.

If no additional provision has been made by Congress to checkmate this move, it is useless to enquire whether the inspecting drug officer of old is still at his post and doing his duty.

This making and selling adulterated drugs is a public crime, for whose full expression language is utterly inadequate. For a dealer, either wholesale or retail, who buys and sells drugs, which he knows to be adulterated, impure or even simply worthless from age or other causes, a prominent place in a Georgia chain gang would be a position far below his distinguished merits.

There is scarcely a State in the Union that has not enacted stringent laws for the protection of its people against the devilsh greed of kerosene and coal oil manufacturers, yet, strange to say, we know of no available statute, anywhere, to protect the same people from the terrible results of a wholesale and retail traffic in adulterated, impure or worthless drugs.

To dispense poison is to destroy life with the certainty of condign punishment at the hands of the sheriff. To dispense inert, unseless drugs, is to destroy human life on a yet larger scale, without fear of a coroner or a jury.

There are other abuses in the drug business, in which physicians are seriously interested, to which we shall, at convenient opportunities, pay our respects.

